# Nogo-A inactivation improves visual plasticity and recovery after retinal injury

**DOI:** 10.1038/s41419-018-0780-x

**Published:** 2018-06-27

**Authors:** Julius Baya Mdzomba, Noémie Jordi, Léa Rodriguez, Sandrine Joly, Frédéric Bretzner, Vincent Pernet

**Affiliations:** 1 CUO-Recherche, Centre de recherche du CHU de Québec and Département d’ophtalmologie, Faculté de médecine, Université Laval, Quebec, QC Canada; 2 Department of Health Sciences and Technology, Brain Research Institute, University of Zurich, ETH Zurich, 8057 Zurich, Switzerland; 3 Centre de recherche du CHU de Québec and Département de psychiatrie et neurosciences, Faculté de médecine, Université Laval, Quebec, QC Canada

## Abstract

Myelin-associated proteins such as Nogo-A are major inhibitors of neuronal plasticity that contribute to permanent neurological impairments in the injured CNS. In the present study, we investigated the influence of Nogo-A on visual recovery after retinal injuries in mice. Different doses of *N*-methyl-d-aspartate (NMDA) were injected in the vitreous of the left eye to induce retinal neuron death. The visual function was monitored using the optokinetic response (OKR) as a behavior test, and electroretinogram (ERG) and local field potential (LFP) recordings allowed to assess changes in retinal and cortical neuron activity, respectively. Longitudinal OKR follow-ups revealed reversible visual deficits after injection of NMDA ≤ 1 nmole in the left eye and concomitant functional improvement in the contralateral visual pathway of the right eye that was let intact. Irreversible OKR loss observed with NMDA ≥ 2 nmol was correlated with massive retinal cell death and important ERG response decline. Strikingly, the OKR mediated by injured and intact eye stimulation was markedly improved in Nogo-A KO mice compared with WT animals, suggesting that the inactivation of Nogo-A promotes visual recovery and plasticity. Moreover, OKR improvement was associated with shorter latency of the N2 wave of Nogo-A KO LFPs relative to WT animals. Strikingly, intravitreal injection of anti-Nogo-A antibody (11C7) in the injured eye exerted positive effects on cortical LFPs. This study presents the intrinsic ability of the visual system to recover from NMDA-induced retinal injury and its limitations. Nogo-A neutralization may promote visual recovery in retinal diseases such as glaucoma.

## Introduction

Retinal degeneration resulting from stroke, diabetic retinopathy, or glaucoma causes irreversible vision loss by inducing neuronal cell death. However, retinal disease models showed that the extent of visual deficits does not always correlate with the level of retinal cell death^1,2^. Functional impairments in surviving neurons may thus account to a large extent for visual deficits after retinal injury. Moreover, the fact that retinal neurons have a very weak ability to regenerate after injury is likely to participate in the vision deterioration caused by ocular diseases^3–5^. Molecular mechanisms restricting visual neuron plasticity have been described in the injured retina. For example, myelin-associated proteins such as Nogo-A inhibit retinal ganglion cell (RGC) axon regeneration after experimental optic nerve injury^6^. The inhibitory effects of Nogo-A are mediated by Nogo-66 receptor 1 (NgR1)^7^ and intracellular RhoA activation^8–10^. The same molecular mechanism may alter vision function after partial retinal lesions.

In intact animals, the visual system plasticity becomes very limited after the end of so-called critical period^11^. For example, the ocular dominance (OD) plasticity in the primary visual cortex (V1) is dramatically reduced after postnatal day 19–32 (P19–32) in mice^12^. The closure of the critical period in the visual system coincides with the formation of myelin and the upregulation of Nogo-A in deep visual cortex layers^13^. Strikingly, genetic ablation of Nogo-A/B or its receptors NgR1 and paired Ig-like receptor B (PirB) restore OD plasticity in the visual cortex of mature mice^13,14^. We observed that the optokinetic reflex (OKR) sensitivity of intact adult Nogo-A knockout (KO) mice was higher than in wild-type (WT) mice^15^ after monocular deprivation (MD)^16^, thus pointing at a major role for Nogo-A in the control of experience-driven visual plasticity^15^. These findings prompted us to test the impact of Nogo-A inactivation on visual recovery after retinal injury in the present study.

Intravitreal injections of *N*-methyl-d-aspartate (NMDA) trigger excitotoxicity in a dose-dependent manner and are therefore suitable to produce various degrees of retinal injuries^17–19^. NMDA-induced excitotoxicity has extensively been used to mimic ischemic-like insults and to study neuronal cell death mechanisms in the rodent retina^20–25^. Surprisingly, the dose-dependent effects of NMDA on the mouse visual system function have not been fully characterized. In the current study, using the model of NMDA-induced excitotoxicity, visual function changes were monitored with the OKR in WT and Nogo-A KO adult mice. Our results revealed the limitations of the visual system to recover from retinal injuries and suggest that neuronal plasticity stimulation with Nogo-A inactivation improves vision after retinal damage ^26–28^.

## Results

### Dose-dependent effects of NMDA on visual loss and recovery

**Fig. 1:**
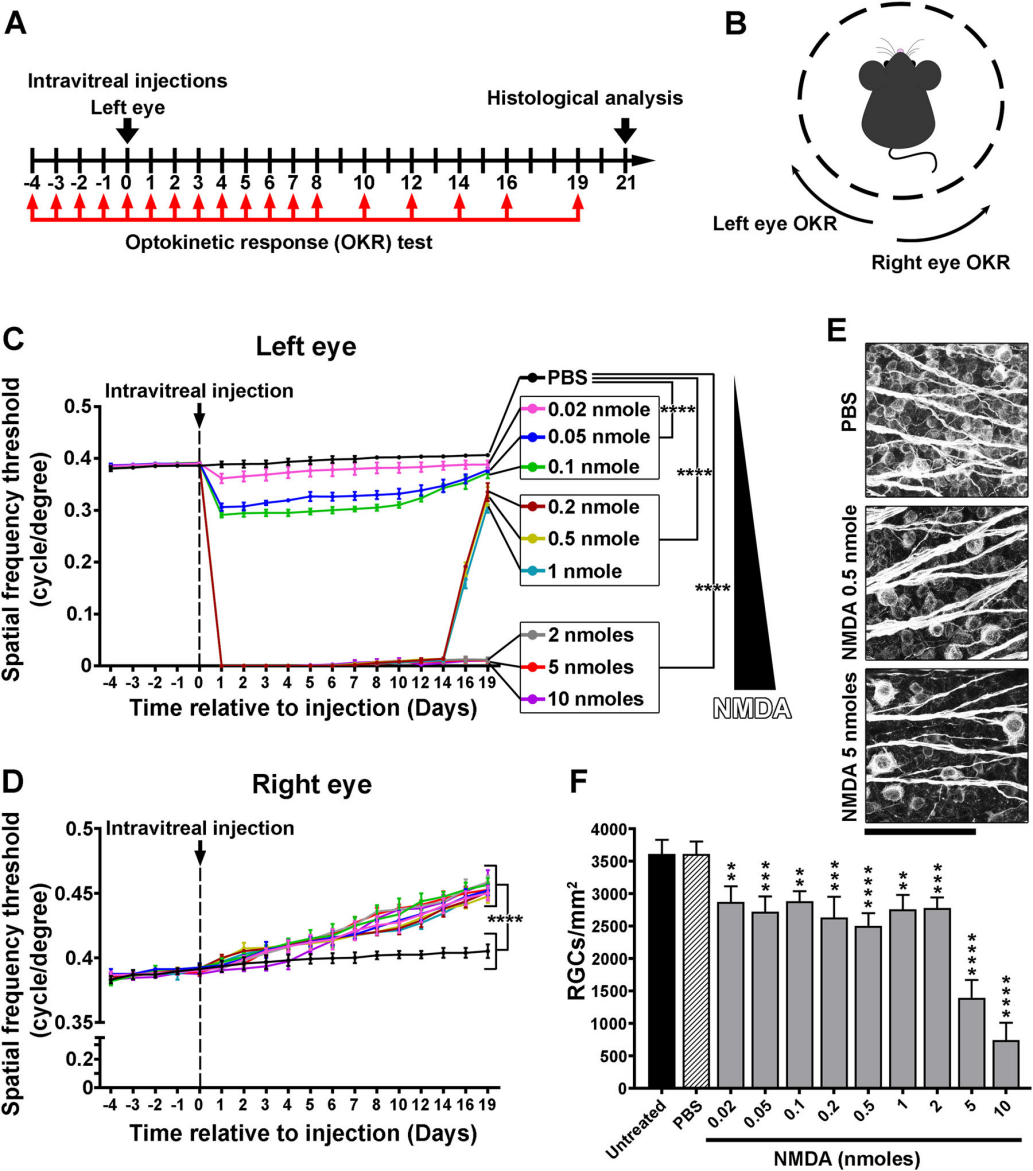
Reversible and irreversible patterns of OKR deficits are induced in a dose-dependent manner by intravitreal injections of NMDA. **a** Experimental timeline of the OKR measurements and histological analysis of RGC survival after the injection of PBS (control) or NMDA in left eye vitreous. **b** Assessment of the left and right eye function with the OKR test. Clockwise and counterclockwise rotation of gratings induced reflexive tracking movements through the left eye or right eye stimulation, respectively. **c** Spatial frequency threshold of the left eye after injection of PBS or NMDA (*n* = 4–6 mice/group, mean ± S.D.). Significant differences were observed between PBS and NMDA treatments from day 1 to day 19 post injection (two-way ANOVA, Tukey’s post hoc test; ****P < 0.0001). **d** Spatial frequency threshold of the non-injected contralateral eye (mean ± S.D.). All NMDA-injected eyes exhibited very significant increase in spatial frequency threshold compared with PBS administration from day 6 on (two-way ANOVA, Tukey’s post hoc test; ****P < 0.0001). **e** Representative images showing the density of surviving RGCs labeled with β3tubulin on retinal flat-mounts. **f** Quantitative analysis of β3tubulin-positive RGC survival (RGC/mm^2^). Each bar represents 3–5 mice/group (mean ± S.D.). The density of cells was calculated in eight areas located in the four retinal quadrants. Statistics: one-way ANOVA followed by Tukey post hoc test, **P < 0.01; ***P < 0.001; ****P < 0.0001. Scale bar in **e** = 100 μm

The optokinetic response (OKR) of adult WT mice was assessed after intravitreal injections of variable concentrations of NMDA into the left eye. Control mice were treated with phosphate-buffered saline (PBS; Fig. 1a). The spatial frequency thresholds of the two eyes were separately evaluated by changing the direction of grating rotation in the virtual-reality optomotor system (Fig. 1b)^29^. PBS injection did not alter the OKR (Fig. 1c). The spatial frequency threshold assessed after NMDA delivery allowed to distinguish three patterns of functional changes: (1) at 0.02–0.1 nmol NMDA—weak visual response reduction and quick and complete recovery; (2) at 0.2–1 nmol NMDA—transient visual response abolition followed by delayed recovery; and (3) at 2–10 nmol NMDA—permanent visual response abolition (Fig. 1c). Surprisingly, visual stimulation of the uninjected contralateral (right) eyes showed enhanced spatial frequency threshold following NMDA injection into the left eye, compared with PBS (Fig. 1d). This phenomenon may result from plastic mechanisms operating in visual brain regions^15,29^. The survival of RGCs was monitored 3 weeks post injection by staining retinal flat-mounts for β3tubulin (Fig. 1a, e, f)^19^. The examination of β3tubulin-labeled RGCs revealed a 30% reduction in cell density after the injection of 0.02–2 nmol NMDA relative to control eyes left untreated or injected with PBS (Fig. 1e, f). A much larger reduction in cell number was observed at 5 and 10 nmol of NMDA at which the decline of RGCs reached ~60% and ~80%, respectively. These data suggest that NMDA-induced visual deficits are sustained for RGC cell death higher than 30%, whereas the elevation of OKR sensitivity through the intact eye did not correlate with retinal lesion severity.

### Endogenous visual recovery after retinal damage

**Fig. 2:**
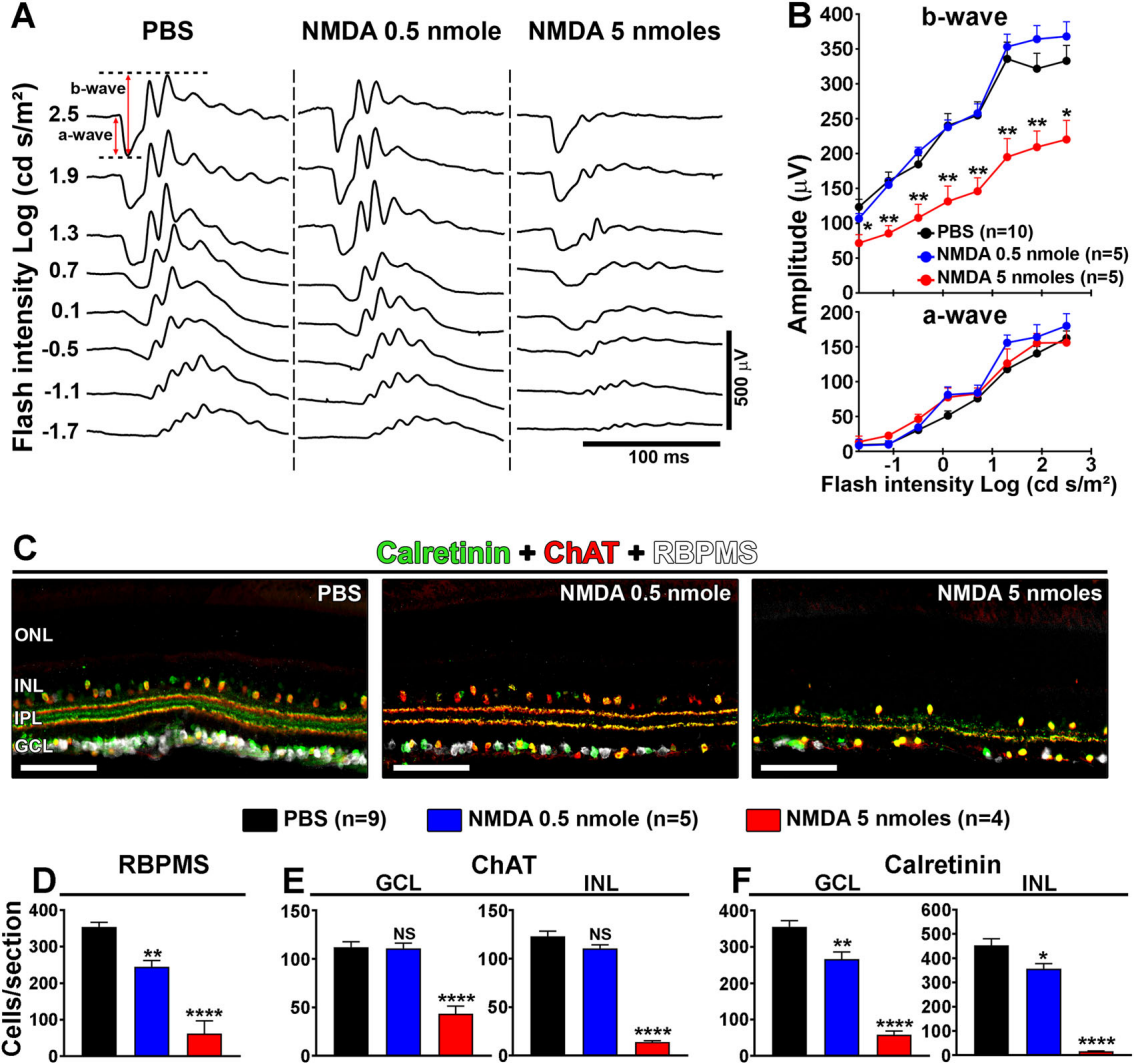
Marked electroretinographic alterations and histological damages are associated with irreversible vision deficits. **a** Representative ERG waveforms recorded at different intensities of light stimulation after PBS and NMDA injection. **b** Quantitatively, ERG b-wave was significantly reduced after intravitreal injection of 5 nmol of NMDA compared with PBS and the administration of 0.5 nmol of NMDA. ERG a-wave amplitudes did not vary. **c** Immunofluorescent stainings of RGCs and amacrine cells, respectively, with RBPMS, ChAT, and Calretinin. **d**–**f** Quantitative analysis of cell survival. The number of RBPMS-labeled RGCs was affected at 0.5 nmol of NMDA. A unit of 5 nmol of NMDA dramatically reduced the number of cells stained with the three markers. Statistics: one-way ANOVA followed by Tukey post hoc test, *P < 0.05; **P < 0.01; ***P < 0.001; ****P < 0.0001. Scale bar in **c** = 100 μm

Amacrine and bipolar cells located in the inner nuclear layer (INL) are also sensitive to NMDA-induced excitotoxicity^17,30–32^. To determine how the activity of these cells changed after reversible or irreversible visual deficits (Fig. 1c), electroretinograms (ERGs) were recorded 10 days after PBS or NMDA injection (Fig. 2a). Indeed, the a-wave of the ERG is generated by the activation of photoreceptors, whereas the b-wave results from the activity of post-photoreceptor cells in the INL. ERG traces did not show differences in a-wave and b-wave amplitudes for 0.5 nmol of NMDA compared with PBS treatment (Fig. 2a, b). However, the b-wave amplitude was decreased by 33–47% after the injection of 5 nmol of NMDA (Fig. 2a, b). Histological analysis allowed to observe RGC and amacrine cell death in the ganglion cell layer (GCL) and in the INL (Fig. 2c). Retinal sections were stained by immunofluorescence for choline acetyltransferase (ChAT), selectively expressed in a subpopulation of amacrine cells (Starburst amacrine cells)^33^, for calretinin, a cell marker found in amacrine subtypes and RGCs^34^ and for RNA-binding protein with multiple splicing (RBPMS), a specific marker of RGCs^35^ (Fig. 2c). After injection of 0.5 nmol of NMDA, the density of cells labeled with RBPMS and calretinin, but not that expressing ChAT, was clearly diminished in the INL and in the GCL. A massive decrease in all labeled cells could be observed at 5 nmol of NMDA. Quantitatively, the number of RBPMS-positive RGCs was reduced by ~30% at 0.5 nmol of NMDA and by ~82% at 5 nmol of NMDA (Fig. 2d). ChAT-labeled amacrine cells were not affected by 0.5 nmol of NMDA but decreased by ~60–90% with 5 nmol of NMDA (Fig. 2e). The rate of cells containing calretinin shrank by ~21–25% after 0.5 nmol NMDA injection and by ~84–96% after delivery of 5 nmol of NMDA (Fig. 2f). Therefore, in contrast to 0.5 nmol of NMDA that has moderate toxic effects, 5 nmol of NMDA leads to marked functional loss and to massive cell death that correlate with irreversible OKR loss.

### Nogo-A is not significantly downregulated in retinal glia after reversible injury

**Fig. 3:**
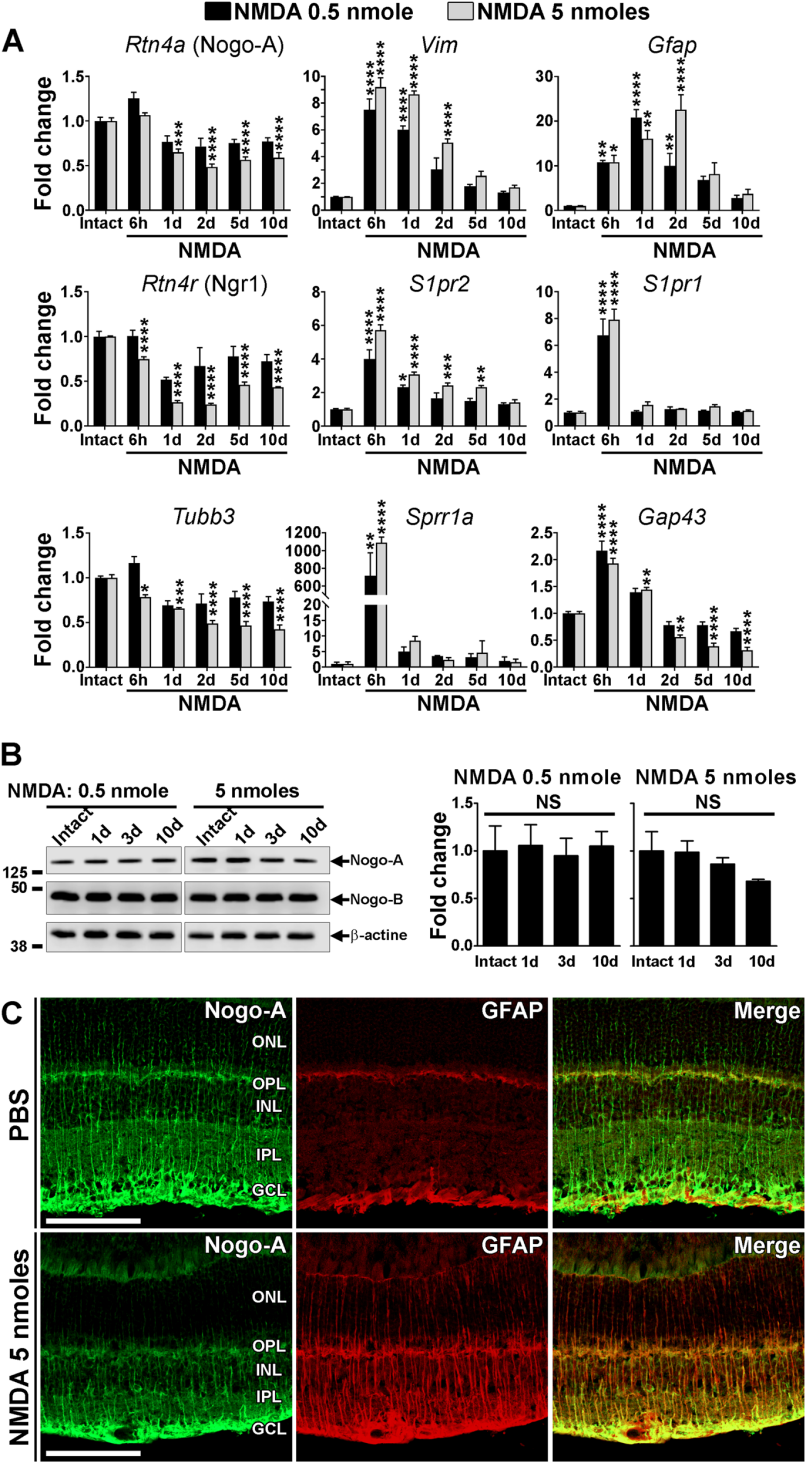
NMDA-induced gene expression changes in the retina. **a** The time-course of gene expression changes was assessed for Nogo-A, gliosis markers (*Vimentin* and *Gfap*), Nogo-A receptors (*NgR1*, *S1pr1*, and *S1pr2*), the neuronal marker β3tubulin (*Tubb3*), and two growth-associated proteins (*Sprr1a* and *Gap43*) by qRT-PCR in retinal lysates. Three to four mice were used for each time point. Statistics: significant changes between intact samples and NMDA-treated retinal samples were evaluated within each dose group using a one-way ANOVA and Tukey’s post hoc tests, *P < 0.05; **P < 0.01; ***P < 0.001; ****P < 0.0001. **b** Western blot analysis for Nogo-A in retinal lysates did not reveal significant fluctuations in protein level after intravitreal injection of 0.5 nmol of NMDA although a trend toward a decrease was observed with 5 nmol (*n* = 3 mice/time point, NS not significant). **c** Retinal sections were stained for Nogo-A and the gliosis marker GFAP 10 days after PBS or NMDA (5 nmol) injection. NMDA induced GFAP upregulation and Nogo-A downregulation in the radial extensions of Müller glia. Scale bar in **b** = 100 μm

The expression of Nogo-A and its receptors was monitored by quantitative real time reverse transcription polymerase chain reaction (qRT-PCR) after excitotoxicity (Fig. 3a). The level of *Nogo-a* (*Rtn4a*) transcripts was only decreased by 5 nmol of NMDA (Fig. 3a). By western blot analysis however, the level of Nogo-A protein did not change (Fig. 3c). Nevertheless, a weaker immunofluorescent signal for Nogo-A was observed in the superior quadrant of the retina treated with 5 nmol of NMDA (Fig. 3b) using 11C7^36^. In the intact retina, Nogo-A signal was intense in Müller cell extensions, consistently with our previous observations^37,39,40,42^. The decreased expression of Nogo-A detected by qRT-PCR coincided with the upregulation of *Glial fibrillary acidic protein* (*Gfap*) and *Vimentin* mRNA (Fig. 3a) and GFAP protein (Fig. 3c), thus excluding the involvement of Nogo-A in Müller cell gliosis^37,43^. Moreover, the expression of the Nogo-A receptor *Ngr1* (*Rtn4r*) was decreased along with that of the neuronal marker *Tubb3* (β3tubulin). In contrast, *Sphingosine 1-phosphate receptor 1 and 2* (*S1pr1* and *S1pr2*) are two potential Nogo-A receptors^44^ whose mRNA levels were upregulated (Fig. 3a). The mRNA elevation of the neuronal growth markers *Growth associated protein 43* (*Gap43)* and *Small proline rich protein 1a* (*Sprr1a*) suggest that endogenous mechanisms of neuronal plasticity may be activated in an attempt to restore retinal function. In this context, the decrease in *NgR1* mRNA may contribute to retinal cell plasticity and visual recovery (Fig. 3a). Paradoxically, *Nogo-a* mRNA downregulation only occurred at a significant level when retinal injury was too severe, i.e., after the delivery of 5 nmol of NMDA, to influence functional recovery and did not change the level of Nogo-A protein (Fig. 3b). The limited downregulation of Nogo-A and NgR1 encountered after moderate injury (0.5 nmol NMDA) may not be optimal for promoting rapid and complete vision recovery.

### Nogo-A neutralization improves visual recovery and plasticity after retinal injury

**Fig. 4:**
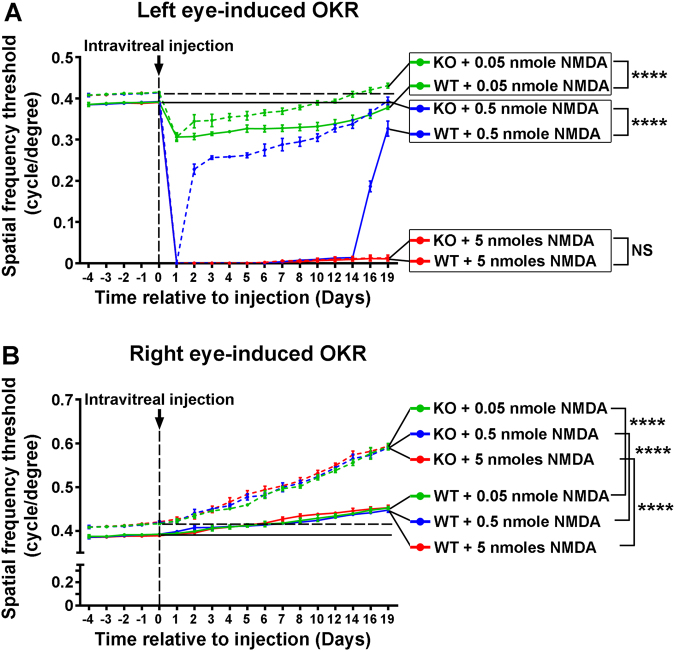
*Nogo-A* gene ablation improves visual recovery and plasticity after NMDA-induced excitotoxicity. **a** The time course of OKR changes was established in WT and Nogo-A KO mice before and after NMDA injection in the left eye (mean ± S.D.). Three doses of NMDA allowed to specifically induce: slight and reversible deficits (0.05 nmol); profound but reversible deficits (0.5 nmol); and complete and permanent deficits (5 nmol). At 0.05 and 0.5 nmol, OKR improved faster in Nogo-A KO mice than in WT mice. Results from WT mice are the same as in Fig. 1. **b** The OKR mediated by right eye stimulation was repeatedly measured to determine visual changes in the non-injured visual pathway. Independently of the dose of NMDA administered, Nogo-A mice showed a stereotyped pattern of spatial frequency threshold improvement compared with WT animals. In all, 4–6 mice were used/group. Statistics from day 1 to day 19 post injection: two-way ANOVA followed by Tukey’s post hoc test, ****P < 0.0001; NS not significantly different

The influence of Nogo-A ablation on the OKR was tested after NMDA injections using Nogo-A KO mice. The OKR of Nogo-A KO mice recovered faster from NMDA-induced injury after the administration of 0.05–0.5 nmol of toxin than in WT controls (Fig. 4a). However, with 5 nmol of NMDA no improvement could be observed. Strikingly, the spatial frequency threshold of the intact (right) eye increased to a higher level in Nogo-A KO mice than in WT mice (Fig. 4b), suggesting that Nogo-A ablation induces functional improvement in the healthy structures of the visual system as well. This phenomenon occurred in a stereotyped manner upon injection, irrespectively of the dose of NMDA administered (Fig. 4b, Fig. S1). Interestingly, the quantification of β3tubulin-labeled cells did not reveal differences in RGC survival between WT and KO mice following NMDA injection (Fig. S1). Therefore, Nogo-A deletion promotes visual recovery and functional plasticity without influencing RGC survival.

### The electrophysiological response of V1 neurons is modified by Nogo-A inactivation

**Fig. 5:**
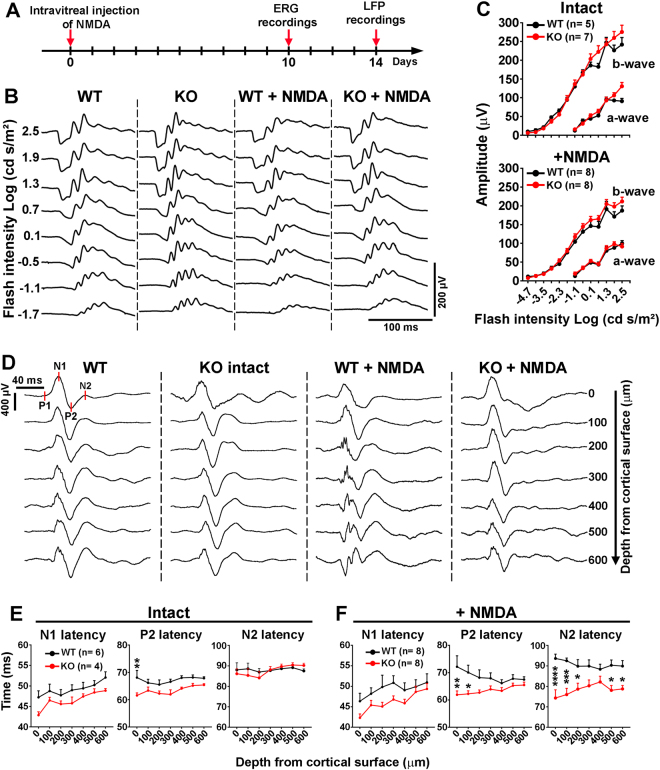
The electrophysiological response of V1 neurons is modified in the absence of Nogo-A. **a** Timeline of ERG and LFP recordings after intravitreal injection. A dose of 0.5 nmol of NMDA was used. **b** ERGs were recorded in WT and Nogo-A KO mice. **c** Quantitative analysis of ERGs did not reveal differences in a-wave and b-wave amplitudes between the two mouse genotypes. **d** LFPs were recorded in the V1 region contralateral to the left injected eye in WT and Nogo-A KO brains. **e** In intact mice, P2 latency was higher in WT than in KO mice. **f** After NMDA-induced retinal lesion, P2 and N2 latencies of WT mice were significantly delayed compared with Nogo-A KO mice. Statistics: two-way ANOVA followed by Bonferroni’s multiple comparison test, *P < 0.05; **P < 0.01; ***P < 0.001; ****P < 0.0001

To examine changes in neuronal activity in the retina and visual brain areas, ERGs and cortical local field potentials (LFPs) were recorded in Nogo-A KO and WT mice (Fig. 5a). The amplitude of the ERG a-wave and b-wave did not significantly differ between the two mouse genotypes with or without NMDA-induced injury, suggesting that the activity of retinal cells, upstream of RGCs, is not modified in the absence of Nogo-A (Fig. 5b, c). We then hypothesized that the visual function improvement induced by Nogo-A deletion may be due to plastic changes in the retinogeniculate pathway and visual brain regions^15^. Neuronal activity was followed in the monocular area of the primary visual cortex (V1) by recording LFPs evoked by light stimulation in the left eye (Fig. 5d, e, f and Fig. S2A, B). LFP waveforms presented characteristic P1–N1 and P2–N2 components (Fig. 5d)^45^ that are thought to respectively stem from primary and secondary retinogeniculate responses^45,46^. In intact animals, only the P2 latency of KO (*n* = 4 mice) LFPs was significantly lower than in WT mice (*n* = 6 mice; Fig. 5d, e and Fig. S2A). In NMDA-injected mice, clearer differences appeared: the latencies of P2 and N2 were shorter in Nogo-A KO (*n* = 8 mice) than in WT (*n* = 8 mice) animals (Fig. 5d, f). These data suggest that cortical activity changes in V1 may participate in the enhancement of visual recovery in Nogo-A KO mice.

**Fig. 6:**
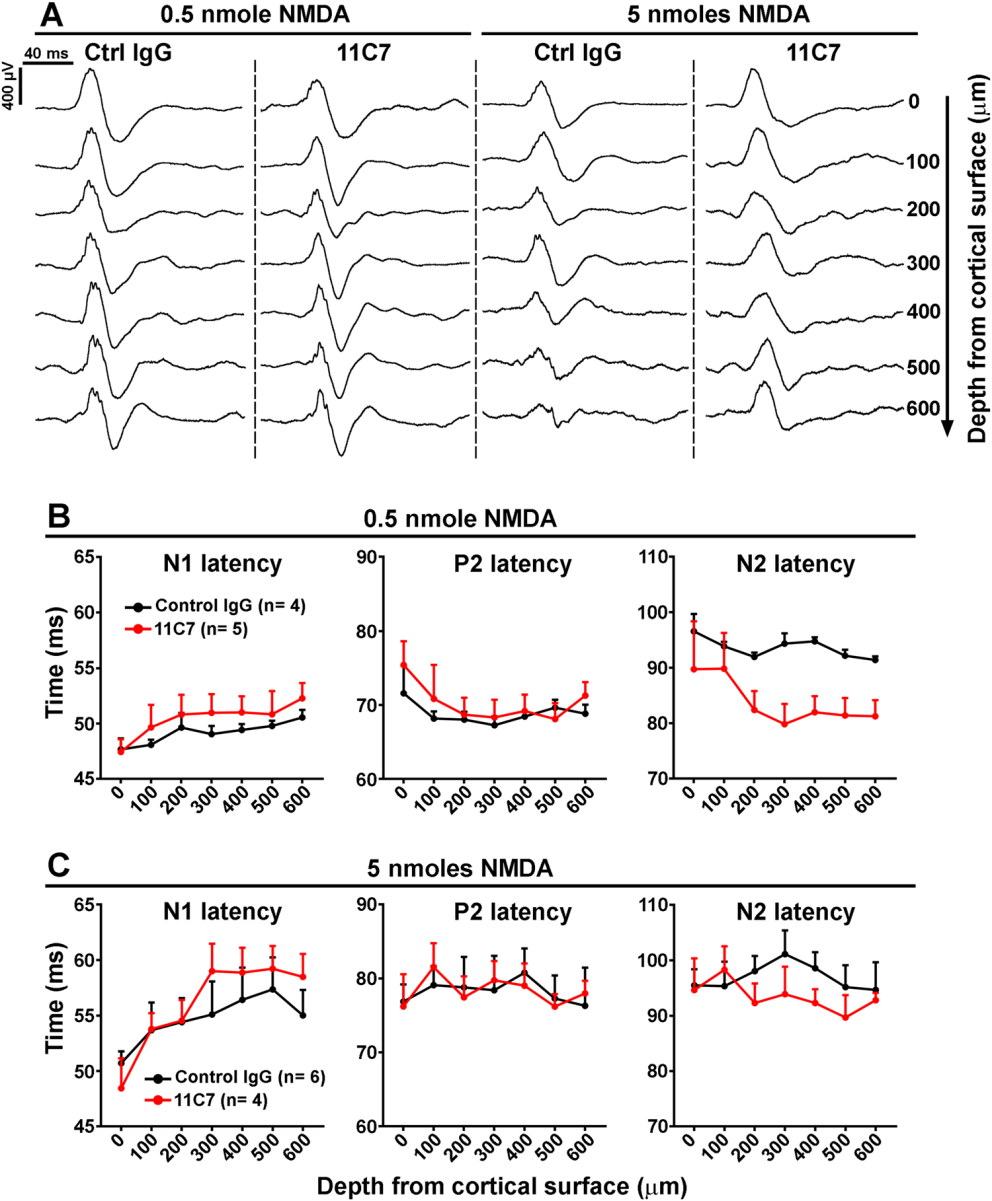
Intravitreal injection of Nogo-A-neutralizing antibody 11C7 changes V1 neuron response after retinal injury. LFPs were recorded in the V1 region contralateral to the left injected eye after intravitreal injection of NMDA followed by the administration of Nogo-A-neutralizing antibody (11C7) or control antibody (Ctrl IgG). Antibodies were injected 2 days after that of NMDA. **a** Representative LFP traces recorded 10 days after NMDA treatments. **b** N2 latencies of mice treated with 11C7 were shorter after retinal lesion induced by 0.5 nmol of NMDA compared with control IgG. However, statistical significance was only reached at depths ranging from 200 to 600 μm with unpaired *t*-test, but not with a two-way ANOVA. **c** However, stronger retinal damage with 5 nmol of NMDA abrogated the effects of 11C7 on N2 latency

To address the potential role of retinal Nogo-A in cortical neuron activation (Fig. 5d, f), the function-blocking antibody 11C7 (2 μg/eye) or a control IgG was intravitreally injected 2 days after NMDA-induced injury (Fig. 6), a time when the expression of inflammation-associated molecules is strongly reduced (Fig. S3). Indeed, 11C7 is efficient to acutely neutralize Nogo-A in the brain^47,48^, spinal cord^49^, and retina^50^. Strikingly, LFP recordings allowed to observe N2 latency reduction with 11C7 treatment compared with control antibody after injection of 0.5 nmol of NMDA (Fig. 6a, b). As 0.5 nmol of NMDA does not alter the ERG waves generated by inner and outer retinal layer cells (Fig. 2a, b), it is plausible that 11C7 modified LFP latencies by influencing RGC function. In contrast, 11C7 had no effect on N2 latency after the administration of 5 nmol of NMDA (Fig. 6a, c). The fact that ERG b-wave (Fig. S4A, B) and RGC survival (Fig. S4C) were similarly affected after delivery of 5 nmol of NMDA and 11C7 or control IgG suggests that 11C7 does not improve the function and survival of retinal cells upstream of RGCs. LFP amplitudes minimally changed after 11C7 treatment (Fig. S2C, D). Collectively, our electrophysiological data reveal that acute blockade of Nogo-A in the eye exerts similar effects on cortical LFPs to those observed in Nogo-A KO mice, presumably by influencing the function of RGCs and their postsynaptic partners.

## Discussion

Our data showed that retinal injuries restricted to the GCL allowed spontaneous visual recovery. The ablation of Nogo-A markedly improved the OKR through the intact and injured eyes and modified cortical LFPs after injury. Acute blockade of retinal Nogo-A with a single injection of 11C7 antibody induced LFP changes as well. As a consequence, Nogo-A appears as a potent inhibitor of vision recovery and plasticity after retinal lesion.

### The intrinsic recovery properties of the injured mouse visual system

NMDA-induced excitotoxicity in the rodent retina is a classical injury model that has been characterized at the anatomical level. Surprisingly, the functional consequences of NMDA-induced excitotoxicity had not previously been analyzed in detail. Here, compared with other experimental models^1,2^, the intravitreal injection of NMDA enabled us to precisely control the extent of retinal injury and to generate measurable OKR dysfunction and recovery. In this context, OKR tests allowed to discriminate three distinct visual alteration patterns: (1) weak visual response reduction with quick and complete recovery for NMDA ≤ 0.1 nmol; (2) transient visual response abolition followed by delayed recovery for 0.2–1 nmol NMDA; and (3) permanent visual response abolition for NMDA ≥ 2 nmol. These results provide new insights on the intrinsic ability of the visual system to recover from retinal injury and its limitations. Our data also reveal visual affection at relatively low dose of NMDA. In general, the use of excessive amounts of NMDA (>2 nmol) may not be optimal to assess the potential benefits of new treatments on visual function impairments. Interestingly, in Nogo-A KO mice, a small (~5% of WT values) but consistent increase in OKR sensitivity was measured before injury, in agreement with our previous results^15^. The difference may result from complex compensatory mechanisms in KO mice but is of modest magnitude when compared with the drastic changes observed between KO and WT mice after NMDA injection.

### The involvement of Nogo-A signaling in the inhibition of retinal neuron recovery

The inhibition of neuronal plasticity and repair induced by Nogo-A may take place in different areas of the visual system, i.e., the optic nerve, the retina, and brain targets, where Nogo-A is constitutively abundant^43^. Nogo-A has been reported to inhibit axonal regeneration in the injured optic nerve of mice^6,37^ by activating NgR1^7^ and RhoA^8^ in RGCs. In addition, Nogo-A is highly expressed in the retina by non-myelinating Müller glia^37^. This specialized kind of glia possesses many cell contacts with neurons and blood vessels^53^. At this level, in the intact retina, Nogo-A may stabilize the neuronal circuits and the vascular network^50^. After injury, however, Nogo-A may exert detrimental effects by limiting neuronal plasticity^6^. In support of this hypothesis, Nogo-A ablation in KO markedly improved OKR recovery. In addition, with a high dose of NMDA (e.g., 5 nmol), the mRNA level of *Nogo-a* was significantly reduced while that of gliosis markers such as *Gfap* and *Vimentin* was upregulated. Therefore, in agreement with our previous results^37^, Nogo-A is unlikely to contribute to gliosis in our model. In addition, the *Nogo-a* mRNA decrease observed by qRT-PCR was not correlated with significant protein downregulation by western blotting. This difference may be due to the relatively long half-life of Nogo-A protein. Although Nogo-A protein was locally decreased around the site of NMDA injection (5 nmol, Fig. 3c), this was insufficient to induce recovery. Moreover, selective retinal Nogo-A blockade with 11C7 injection in the eyeball reduced the N2 latency of cortical LFPs in a similar fashion to chronic Nogo-A ablation in KO animals. This change suggests that retinal Nogo-A impairs cortical neuron activation in V1, perhaps by inhibiting the plasticity of RGC projections in brain targets. For example, in non-injured Nogo-A KO mice, histological rearrangements of retinogeniculate projections were observed in the MD paradigm^15^. LFP recordings did not allow to directly evaluate retinogeniculate dysfunction in our experiments, however. Indeed, although a delay in N1 latency can reflect electrical conductance defects resulting from retinogeniculate projection demyelination or hypomyelination^54–56^, NMDA-induced RGC injury did not produce such changes (Fig. 5d–f)^45^. Additional anatomical examinations of retinotectal projections may help determine if Nogo-A inactivation promotes retinal projection remodeling in the brain. In general, local inactivation of Nogo-A in the retina, in the lateral geniculate nucleus and in V1 may clarify the role of each structure in visual recovery.

### Nogo-A inactivation enhances visual brain plasticity

Our OKR behavior tests and LFP recordings indicate that Nogo-A impedes neuronal activation in brain structures after retinal injury. Previous studies involved Nogo-A in the inhibition of cortical neuron plasticity in different regions of the intact cerebral cortex^13,14,57^. The visual cortex was shown to participate in MD-induced OKR enhancement in intact Nogo-A KO mice^15^. Similarly to what has been observed in the motor cortex^58^, Nogo-A inactivation may facilitate functional synapse formation in V1 pyramidal cells and compensate for retinal damage. A role for Nogo-A in V1 activation is supported by the shorter latency of the P2–N2 component of KO LFPs after NMDA-induced injury. Moreover, Nogo-A KO mice presented a stronger increase in right eye OKR than WT controls upon NMDA injection in the left eye. This phenomenon was independent of the doses of NMDA, including those that weakly affected left eye-driven OKR. Therefore, the intact eye-mediated OKR enhancement cannot simply be viewed as a compensatory mechanism induced by visual deficits in the left eye, contrary to what is observed after MD^13,15^.

### The role of glial vs neuronal Nogo-A on vision recovery and plasticity

Systemic *Nogo-A* gene ablation in KO mice resulted in visual function improvement after retinal damage. However, Nogo-A deletion in neurons may attenuate visual recovery and plasticity enhancement at the same time. Indeed, neuronal Nogo-A positively contributed to the growth response of injured RGCs after optic nerve injury^37^. However, the role of neuronal Nogo-A is not clear in the model of NMDA-induced excitotoxicity. Contrary to optic nerve injury, NMDA injection did not increase the level of Nogo-A in RGCs (Fig. 3). This difference may be due to the severity, the type, and the site of injury: optic nerve crush is a complete traumatic injury inflicted at the axonal level, whereas NMDA (e.g., 0.5 nmol) can produce partial injuries by activating excitotoxic mechanisms in RGC bodies. In this later injury mode, visual recovery may involve compensatory fiber sprouting from spared neurons, similarly to what has been described after spinal cord injury^59^. In the future, the relative contribution of neuronal vs glial Nogo-A should be addressed using conditional KO mouse lines^6^. The main drawback of targeting Nogo-A-specific exon(s), at least in our hands, is the compensatory upregulation of proteins such as Nogo-B, a spliced variant of Nogo-A, in Nogo-A KO brains^6,60^. Indeed, Nogo-B expression is strongly increased in many brain regions but not in the retina^6,37^. A retinal contribution of Nogo-B to visual recovery/plasticity seems therefore unlikely but cannot be excluded. In the Nogo-A KO visual cortex, increased Nogo-B expression is not expected to influence much neuronal plasticity as Nogo-B has a very low affinity for NgR1^61^, a receptor through which Nogo-A inhibits neuronal growth^62^. Moreover, the deletion of Nogo-A/B or that of NgR1 enhances cortical neuron plasticity in V1 to a similar extent, suggesting that Nogo-A predominantly inhibits V1 neuron plasticity via NgR1 activation^13^. Alternatively, acute neutralization of Nogo-A with function-blocking antibodies or viral vectors may allow to clarify the effects of glial Nogo-A on the visual system plasticity.

### The modulation of neuronal plasticity in ocular disease treatments

Our data prompt us to push forward the notion that neuronal plasticity activation could promote vision recovery in ocular diseases. Our data showed that independently of neuronal survival, deleting Nogo-A increased visual recovery and changed the cortical response to light stimulation. In a similar manner, intravitreal injection of the function-blocking antibody 11C7 was able to modify cortical LFPs. We attribute these effects to functional changes that may occur in surviving RGCs and their postsynaptic neuronal partners in the brain since 0.5 nmol of NMDA did not affect the ERG response generated by inner and outer nuclear layer cells. In fact, anti-Nogo-A antibodies may only be efficient to treat ocular diseases whose deleterious effects are limited to RGCs, such as glaucoma. Further investigations will be needed to establish the effects of 11C7 on mouse vision with behavioral tests such as the OKR. Given the high expression of Nogo-A throughout the visual system, the development of ocular treatments based on Nogo-A inhibition will also require to specify how intravitreal vs intracerebral delivery influences vision.

## Conclusion

This study revealed that neutralizing Nogo-A exerts beneficial effects on visual recovery and plasticity after retinal injury. Based on our results, new immunotherapies against Nogo-A may be designed to treat visual disorders.

## Materials and methods

### Animals

Adult male C57BL/6J Nogo-A KO and age-matched C57BL/6J WT mice^60^ were used for behavioral and electrophysiological measurements and tissue analysis. Nogo-A KO animals have previously been described^60^. WT and Nogo-A KO mice originate from two colonies sharing the same C57BL/6J genetic background. Indeed, Nogo-A KO mice were bred with C57BL/6J WT mice for more than 20 generations at the Brain Research Institute of Zurich, Switzerland, so that WT and Nogo-A KO mice used in this study had the same genetic background. C57BL/6J mice treated with antibodies were purchased from the Jackson Laboratory. Animal experiments were carried out in accordance with the guidelines of the Canadian Council on Animal Care, of the Université Laval Animal Welfare Committee and of the Cantonal Veterinary Office in Zurich.

### Intraocular injections

To injure the retina, 2 µl of NMDA (0.01–5 mM) were intravitreally injected in the left eye with a 10-μl Hamilton syringe (Syringe 10UL Model 701RN, Fisher Scientific, Toronto, ON, Canada) under general anesthesia with isoflurane. To do this, the needle tip was inserted through the sclera into the dorsal quadrant of the eyeball, next to the limbus. Care was taken not to injure the lens. The needle tip was kept in place for 2–3 min after injection to allow NMDA diffusion in the vitreous, and slowly withdrawn to limit backflow. The site of injection was then sealed with surgical glue (Histoacryl^®^, Braun). For acute Nogo-A blockade, the same injection procedure was used to deliver 2 µl of 11C7 (1 µg/µl), a function-blocking antibody directed against Nogo-A, 2 days after NMDA injection. 11C7 selectively neutralizes the inhibitory properties of Nogo-A by binding its Δ20 domain^36^. It has been proven efficient at enhancing long-term potentiation in the hippocampus^63^ and at increasing neuronal plasticity in the injured brain^47,48^ and spinal cord^49^. An antibody recognizing ethyl *N*-(3,5-dicarboxyphenyl)-P-{*N*-[5′-(2″,5″-dioxo-1″-pyrrolidinyl) oxy-1′,5′-dioxopentyl]-4- aminophenylmethyl} phosphonamidate was used as a control.

### OKR test

The virtual optomotor system (OptoMotry; Cerebral Mechanics, Middlesex, UK) developed by Prusky et al.^16,29^ was used to evaluate the spatial frequency threshold of the OKR in freely moving mice. In brief, mice were placed on an elevated platform in the middle of an arena surrounded by four computer screens while moving gratings of different spatial frequencies were displayed on the monitors. The reflexive tracking movement of the mouse head in the temporal-to-nasal direction allowed to test the two eyes independently by changing the direction of the visual stimulus^15,29^.

### ERG recording

ERGs were recorded with a Ganzfeld ERG system (Phoenix Research Labs, Pleasanton, CA, USA) as described in our previous study^64^. Scotopic ERGs were recorded after overnight dark adaptation. Under dim red light, mice were anesthetized with an intraperitoneal injection of ketamine (10 mg/kg) and xylazine (1 mg/kg). Pupils were dilated with a drop of 1% Mydriacyl Tropicamide (Alcon). To prevent corneal desiccation and to establish contact between the cornea and the electrode (gold-plate objective lens), a moisturizing solution of 2.5% hypromellose (Goniosoft, OCuSOFT Inc.) was applied right after the beginning of anesthesia. ERGs (bandwidth: 2–1000 Hz) were obtained in response to increasing flash intensities, ranging from −1.7 to 2.5 log cd s m^-2^ (interstimulus interval, 20 ms; flash duration, 1 ms; 0.6 log-unit increment). For quantitative analysis, the amplitude of the a-wave was measured from baseline to the most negative trough, whereas that of the b-wave was measured from the trough of the a-wave to the most positive peak of the retinal response^65^. Statistical analysis was performed using a one-way analysis of variance test followed by Tukey post hoc test (GraphPad Prism, GraphPad Software, La Jolla, CA, USA).

### LFP recording in the visual cortex

Mice were anesthetized with an intraperitoneal injection of ketamine (10 mg/kg) and xylazine (1 mg/kg). In short, animals were placed in a stereotaxic frame and the skin was longitudinally incised to expose the skull. A hole of ~0.5 mm in diameter was drilled in the skull at the stereotaxic coordinates of the monocular primary visual cortex (V1, 3.6 mm caudal to bregma and 2.3 mm lateral) in the right cerebral hemisphere. Bone fragments were removed and exposed brain was covered with a drop of mineral oil to prevent desiccation. A silver ground wire was placed in the muscles below the skull and a tungsten recording electrode was lowered in V1. The left eye was stimulated with light flashes (2.12 Klux, 1 ms, 7 s interstimulus interval) emitted by a light-emitting diode placed at 5 mm from the cornea. Twenty LPF recordings were averaged for each depth starting from the cortical surface up to 600 μm. The signals were amplified (gain ×1000) and band-pass-filtered (10–1000 Hz) using a differential AC amplifier (A-M systems Micro Electrode amplifier model 1800). Signals were digitally converted (Digidata 1440A, Axon Instruments) and analyzed with pClamp 10 (Molecular Device). LFP parameters were analyzed following the description of Creel et al.^45^. Consequently, the latency and amplitude of N1, P2, and N2 were compared between WT and Nogo-A KO mice on one hand and between 11C7 and control IgG treatment on the other hand.

### RGC survival analysis on retinal flat-mounts

RGC survival was estimated 3 weeks after NMDA injection on retinal flat-mounts^2,37,66^. In brief, flat-mounted retinae were washed with 0.1 M PBS and permeabilized with 100% methanol for 20 min at −20 °C. Retinal flat-mounts were immunofluorescently labeled for β3tubulin, a marker of RGCs. In this aim, retinae were incubated with a rabbit anti-β3tubulin antibody (1:500, Abcam, Cambridge, UK, #ab18207) diluted in blocking solution (PBS containing 5% bovine serum albumin (BSA), 0.3% Triton X-100, and 0.05% sodium azide) and then with a secondary anti-rabbit antibody. Images were taken with a Leica SPE-II confocal microscope at ×40. RGCs were counted in regions of 62 500 µm^2^ at 1 and 1.5 mm from the optic disk in the four retinal quadrants.

### Retina processing and immunostaining

Mice were euthanized with an overdose of anesthetics (ketamine 90 mg/kg and xylazine 10 mg/kg) and perfused intracardially with PBS and 4% paraformaldehyde (PFA). Eyes were dissected by removing the cornea and the lens from the eyecup. The eyecups were post-fixed in 4% PFA overnight at 4 °C. The tissues were then cryoprotected in 30% sucrose and frozen in OCT compound (Tissue-TEK, Cedarlane, Burlington, ON, Canada) with a 2-methylbutane bath cooled with liquid nitrogen. Retinal sections were cut (14 μm) with a cryostat. Immunohistochemical stainings were performed in a blocking solution (5% BSA and 0.3% Triton X-100 in PBS). Primary antibodies were applied overnight at 4 °C and after PBS washes, sections were incubated with the appropriate secondary antibody for 1 h at room temperature. The slides were mounted in Vectashield solution (BioLynx, Brockville, ON, Canada). Primary antibodies were as follows: rabbit anti-RNA-binding protein with multiple splicing (1:200, PhosphoSolutions, Aurora, CO, USA, #1830-RBPMS); goat anti-choline acetyltransferase (1:100, Cedarlane, #AB144P); mouse anti-calretinin (1:500, Swant, Marly, Switzerland, #6B3); rabbit anti-GFAP (1:500, Agilent Technologies Canada Inc., Mississauga, ON, Canada); mouse anti-Nogo-A (1:200, Novartis); and rabbit anti-β3tubulin (1:1000, Abcam, #ab18207).

### Semi-qRT-PCR

After cervical dislocation, retinae were rapidly dissected, placed in eppendorf tubes, flash frozen in liquid nitrogen, and stored at −80 °C until RNA extraction. Total retinal RNA was prepared using the RNeasy isolation kit (Qiagen, Toronto, ON, Canada), including a DNase treatment to digest residual genomic DNA. For reverse transcription, equal amounts of RNA were transformed by oligo(dT) and M-MLV reverse transcriptase (Fisher Scientific). Ten nanograms of cDNA were amplified in the Light Cycler 480 thermocycler (Roche Diagnostics Canada, Laval, QC, Canada) with the SYBR Green I Master polymerase ready mix (Roche Diagnostics Canada). The appropriate primer pairs were designed to span intronic sequences or to cover exon–intron boundaries (Table 1). Relative quantification was calculated using the comparative threshold cycle (ΔΔCT) method. cDNA levels were normalized to *Gapdh* as a reference gene, and a control sample (calibrator set to 1) was used to calculate the relative values. Each reaction was done in triplicate, and three to four mice per condition were analyzed.

**Table 1:** Primer sequences used for qRT-PCR measurements in mouse retinal lysates after intravitreal injections of NMDA.

Gene names	Forward (5′–3′)	Reverse (5′–3′)	Product (bp)
*Cox2*	GACAGATCATAAGCGAGGAC	TACACCTCTCCACCAATGAC	153
*Cntf*	CTCTGTAGCCGCTCTATCTG	GGTACACCATCCACTGAGTC	125
*Gapdh*	CAGCAATGCATCCTGCACC	TGGACTGTGGTCATGAGCCC	96
*Gap43*	TGCTGTCACTGATGCTGCT	GGCTTCGTCTACAGCGTCTT	127
*Gfap*	CCACCAAACTGGCTGATGTCTAC	TTCTCTCCAAATCCACACGAGC	240
*Il6*	ACCGCTATGAAGTTCCTCTC	CTCCGACTTGTGAAGTGGTA	163
*Lif*	AATGCCACCTGTGCCATACG	CAACTTGGTCTTCTCTGTCCCG	216
*Rtn4 (Nogo-A)*	CAGTGGATGAGACCCTTTTTG	GCTGCTCCTTCAAATCCATAA	90
*Rtn4r (NgR1)*	CTCGACCCCGAAGATGAAG	TGTAGCACACACAAGCACCAG	116
*S1pr1*	TCAGGGAACTTTGCGAGTGA	AACAGCAGCCTCGCTCAAG	123
*S1pr2*	CATCGCCATCGAGAGACAAG	TCAGACAATTCCAGCCCAGG	146
*Sprr1a*	GAACCTGCTCTTCTCTGAGT	AGCTGAGGAGGTACAGTG	91
*Stat3*	CAAAACCCTCAAGAGCCAAGG	TCACTCACAATGCTTCTCCGC	139
*Tnfa*	CCACGCTCTTCTGTCTACTGA	GGCCATAGAACTGATGAGAGG	92
*Tubb3*	GGCCTCCTCTCACAAGTATG	TTGCCAGCACCACTCTGAC	138
*Vim*	TACAGGAAGCTGCTGGAAGG	TGGGTGTCAACCAGAGGAA	113

## Electronic supplementary material

FIGURE S1

FIGURE S2

FIGURE S3

FIGURE S4

Supplementary figure legends
