# Supplementary figures and images for: Nogo-A inactivation improves visual plasticity and recovery after retinal injury
Source: Cell Death Dis. 2018 Jun 27;9(7):727. doi: 10.1038/s41419-018-0780-x (PMC6021388; doi:10.1038/s41419-018-0780-x)

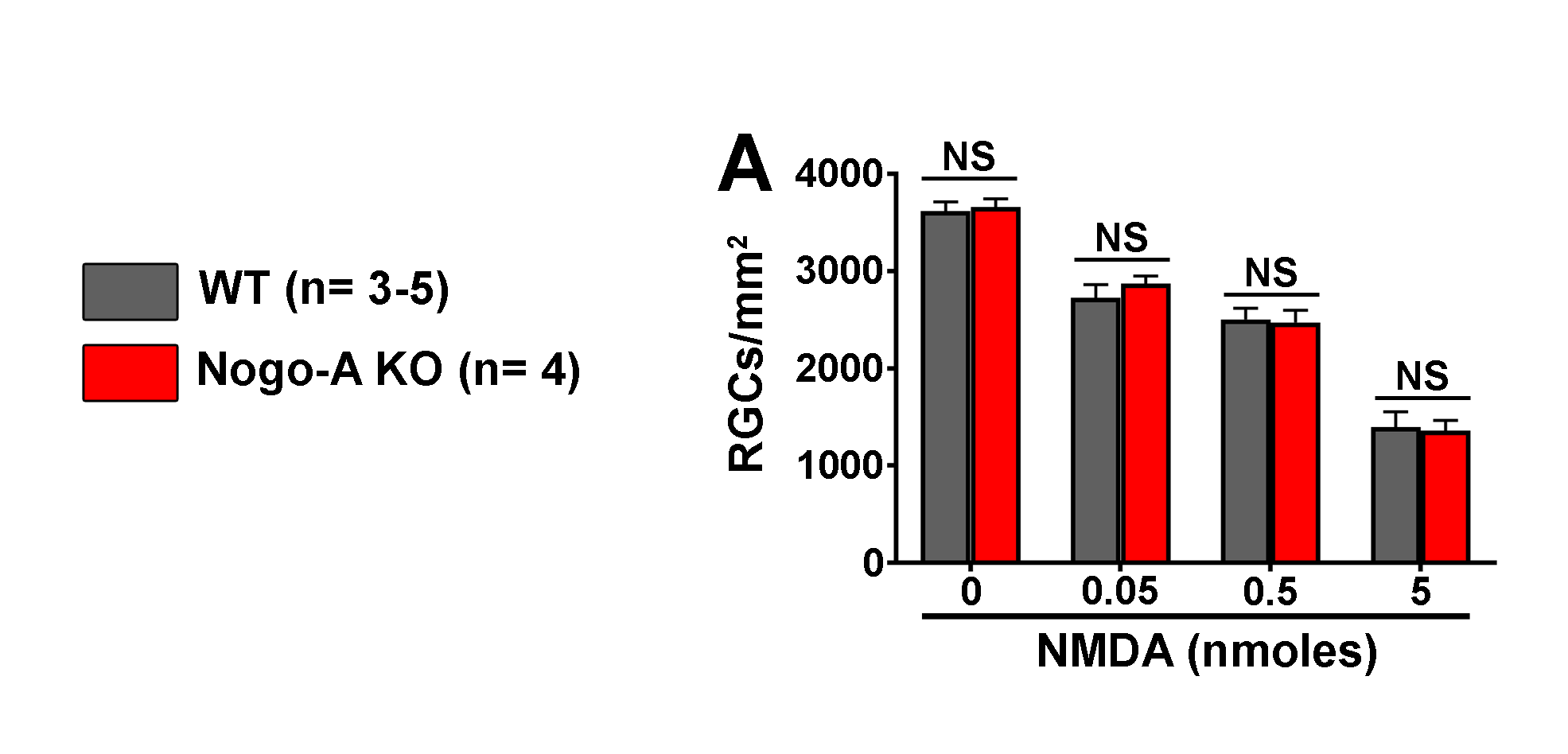

Supplement: Supplementary file 1 — FIGURE S1 [file 41419_2018_780_MOESM1_ESM.tif]

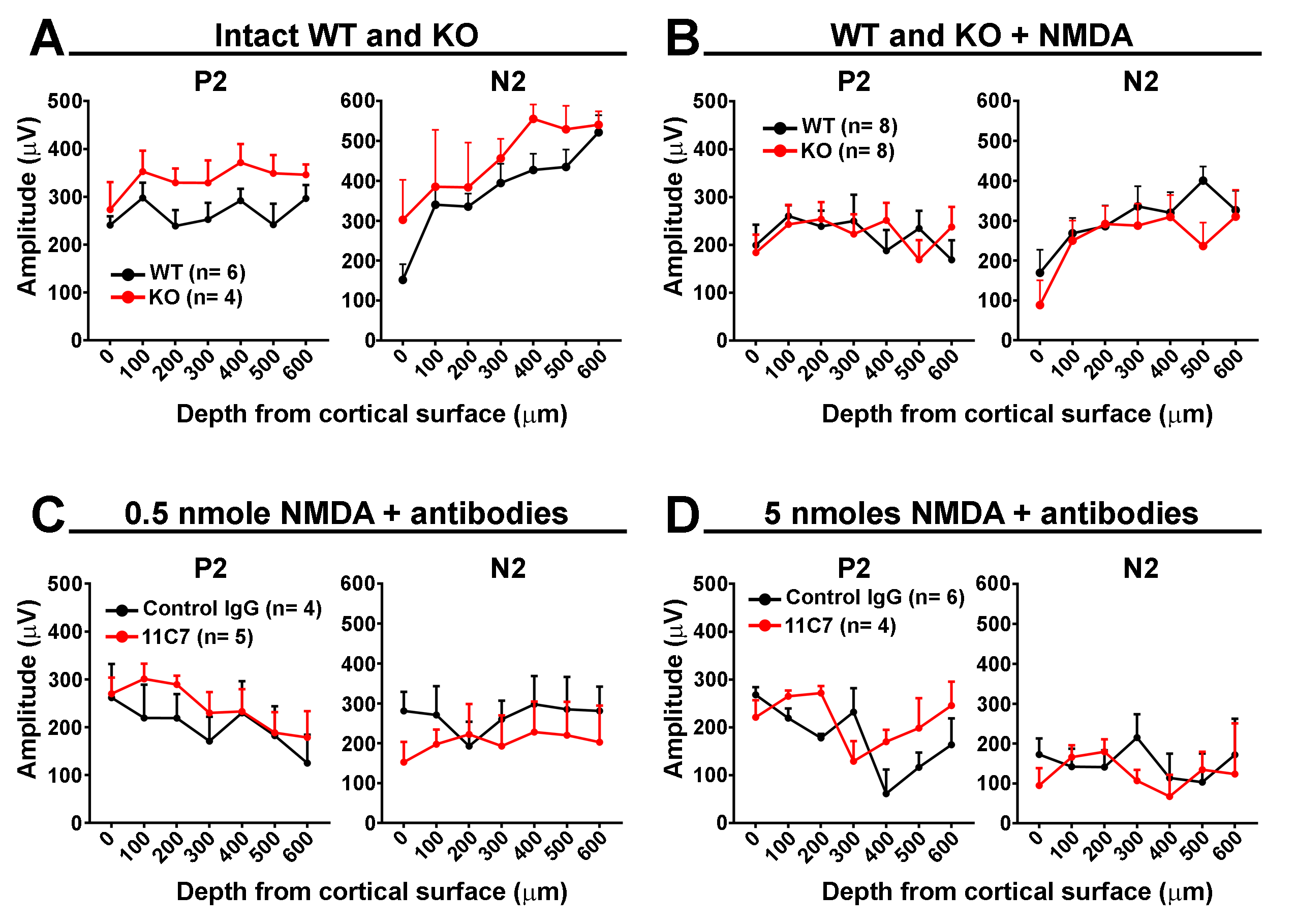

Supplement: Supplementary file 2 — FIGURE S2 [file 41419_2018_780_MOESM2_ESM.tif]

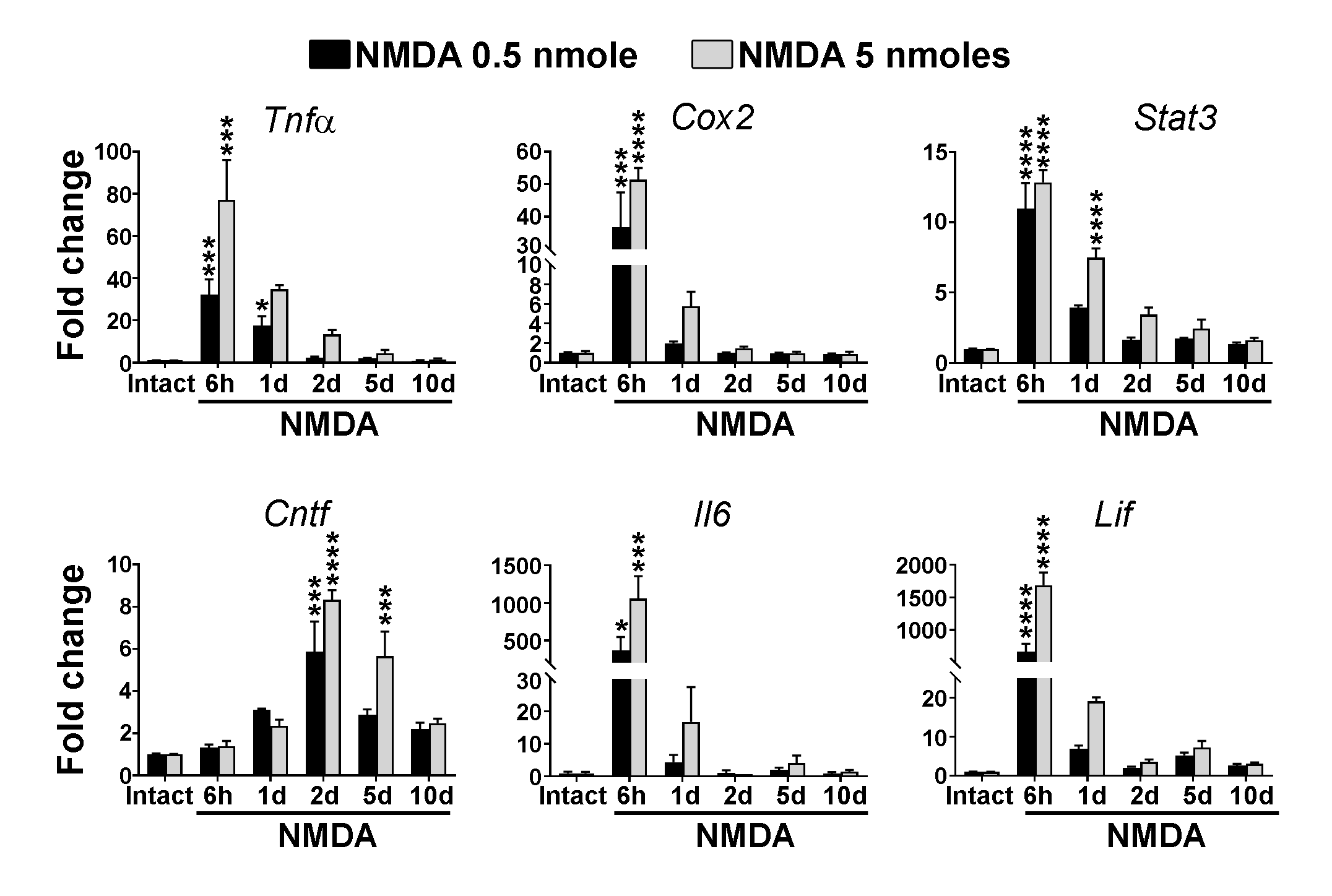

Supplement: Supplementary file 3 — FIGURE S3 [file 41419_2018_780_MOESM3_ESM.tif]

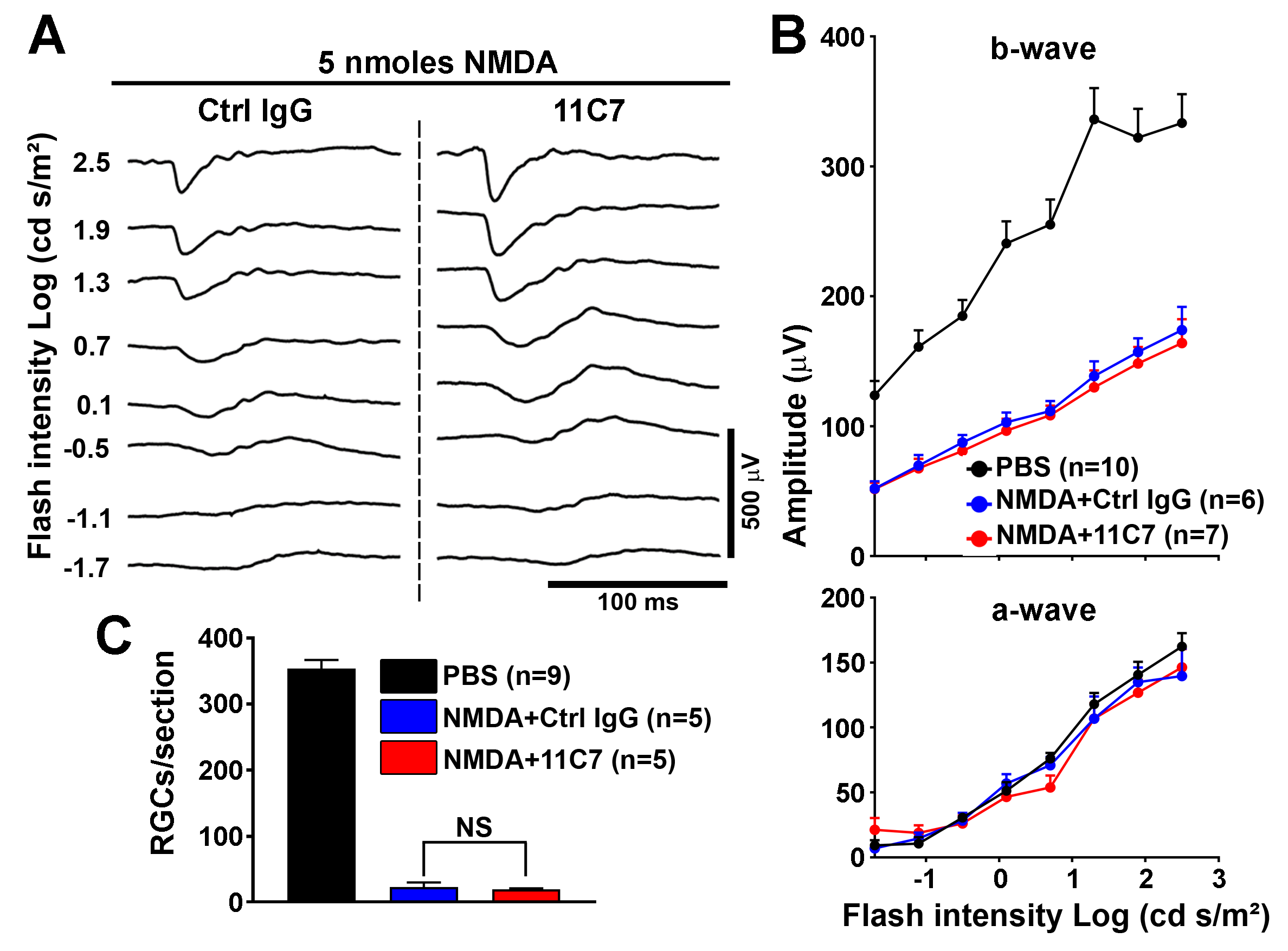

Supplement: Supplementary file 4 — FIGURE S4 [file 41419_2018_780_MOESM4_ESM.tif]
