# Supplementary material for: Nogo-A inactivation improves visual plasticity and recovery after retinal injury
Source: Cell Death Dis. 2018 Jun 27;9(7):727. doi: 10.1038/s41419-018-0780-x (PMC6021388; doi:10.1038/s41419-018-0780-x)
Supplement: Supplementary file 5 — Supplementary figure legends [file 41419_2018_780_MOESM5_ESM.doc]

**Figure S1. RGC survival quantification on retinal flat-mounts.** The average number of β3Tubulin-positive RGCs was calculated in whole retinal flat-mounts after NMDA injections followed by OKR measurements (see Fig. 4). The density of RGCs was not different between WT and Nogo-A KO eyes. NS= not significantly different.

**Figure S2. Comparison of LFP wave amplitudes between WT and Nogo-A KO mice and after antibody delivery. A** The amplitudes of P2 and N2 were measured in the right V1 after left eye stimulation with light flashes. Recordings were carried out at different depths of V1 ranging from the cortical surface (0 μm) until 600 μm. Nogo-A deletion did not affect N2 and P2 amplitudes in KO when compared with WT mice. **B** No difference in N2 and P2 amplitudes appeared between the 2 mouse groups after intravitreal injection of NMDA (0.5 nmole), except for N2 at 500 μm. **C** Intravitreal injection of 11C7 did not change P2 and N2 amplitudes after excitotoxicity induced with a NMDA dose of 0.5 nmole. **D** The amplitude of P2 was only increased by 11C7 at a depth of 200 μm from the cortical surface relative to ctrl IgG.

**Figure S3. Inflammation gene expression after intravitreal injection.** The gene expression of inflammation-associated molecules was determined by qRT-PCR in retinal lysates treated with 0.5 or 5 nmoles of NMDA (n = 3-4 mice/group). NMDA induced the gene upregulation of *Tnfα*, *Cox2*, *Stat3*, *Il6* and *Lif* up to 24 h post NMDA administration, except *Cntf* whose mRNA level increase was higher than in intact samples at 2 d and 5 d after the injection of 5 nmoles of NMDA. Statistics: One-way ANOVA followed by Tukey post hoc test, *:P<0.05; **:P<0.01; ***:P<0.001; ****: P<0.0001.

**Figure S4. Retinal function and RGC survival analyses after intravitreal injection of NMDA and antibodies. A** ERGs were recorded in mice injected with 5 nmoles of NMDA and treated with 11C7 or control IgG antibody. Representative ERG traces were obtained 10 days after the injection of NMDA. **B** Luminance-response curves for the a-wave and b-wave did not show amplitude difference between 11C7 and control IgG treatments for variable intensities of light stimulation. The values of mice injected with PBS (same results as in Fig. 2B) were shown on the same graph for the sake of comparison. **C** The survival of RBPMS-labelled RGCs did not differ between the two mouse groups treated with antibodies (mean ± S.D., unpaired t-test, NS= not significantly different).
